# Exsection of the Superior Maxillary, Together with the Malar and Palate Bones of the Right Side. Recovery

**Published:** 1860-07

**Authors:** W. J. Leake

**Affiliations:** Yazoo City, Miss.


### ARTICLE XII.

*Exsection of the Superior Maxillary, together with the Malar and Palate Bones of the Right Side. Recovery.* By W. J. LEAKE, M. D. of Yazoo City, Miss.

THE subject of this operation was a negro man, aged about 25 years, the property of the Hon. A. P. Hill, of Madison county. He was placed under my charge about the 1st June last. At that time a large tumor occupied the right side of the face, the right eye being much protruded, the right nostril entirely obstructed by the tumor, which had also dislocated the os palati of that side, together with the corresponding palatine process of the maxilla along the posterior two-thirds or three-fourths of the mystachial suture—the anterior part of the suture being intact. The molar and bicuspid teeth of the right side had been removed some time previously. A foul discharge issued from the right nostril, and from an opening in the alveolar aspect of the tumor.. The opening would have admitted the end of the little finger, being partly occupied by a tough, white substance, which came away in a few days, and proved to be a portion of the periosteum which had lined the antrum. The end of a silver male catheter was passed about an inch up the opening. The patient was much emaciated, and so much debilitated that his gait was tottering and unsteady. The pulse was feeble and frequent. His mental condition approached imbecility; appetite impaired. I have not been able to get a satisfactory history of the case previous to the time it fell under my observation. As nearly as I could ascertain, the disease commenced some two years previously, at which time the patient complained of great pain in the teeth and jaw. The teeth were extracted, but no relief obtained. When brought to me he did not complain of much pain.

For the relief of the patient, an operation consisting in the entire removal of the tumor was clearly demanded; but his condition at the time forbade any surgical procedure. He was, therefore, put upon a tonic, cordial treatment, with a nutritious diet, in order to prepare him for an operation at a future time. The local treatment consisted of detergent and astringent injections of the right nostril, and opening in the tumor. Under this treatment he gradually improved; the opening in the tumor filled up by granulations; and by closing the left nostril and mouth, and making a forcible expiration, air passed through the right nostril with a hissing sound; but a probe could not be passed.

The patient having improved sufficiently, the operation was performed on the 18th of August, and was in nearly all its steps the one advised by Mr. Liston. My partner, Dr. Barnett, and Drs. Kidd, Wilson, and Peake, were present. A cut, commencing near the inner angle of the eye, was extended down the side of the nose, beneath the ala, to the ridge bounding the sulcus of the lip, making the division of the lip through that ridge. Another cut, commencing at the same point with the first, was carried beneath the eye, in a line with the fibres of the orbicularis muscle, to a point corresponding to the transverse facial suture. The ala of the nose was next cut up from its connection with the tumor, and the soft parts dissected off the tumor and turned back. In order to command the tumor posteriorly, it was found necessary to make an additional cut, commencing at the terminus of the last, and extending along the line of the zygomatic process of the malar bone, something over an inch. The lateral incisor of the right side having been extracted by H. Lawrence, D. S., I proceeded to notch the alveolar process with Liston's cutting pliers, and to divide the palatine process through the roof of the mouth. The nasal process was then severed, followed by the division of the zygomatic process of the malar bone, and, lastly, the malar and frontal bones were severed through the transverse suture. The bony connections of the tumor were next all separated. The forceps were now applied, one branch in the orbit, the other in the mouth, and an attempt made to dislodge the tumor. In this, however I failed, the tumor crushing under the pressure of the instrument. It was found necessary to pass a broad chisel behind the tumor, after which it was dissected out with the knife and curved scissors. The lips of the wound were brought together, and secured by the interrupted suture, except through the lip, which was secured by two silver pins. The cheek was stuffed with a silk handkerchief.

Very little blood was lost during the operation. Not exceeding f℥xvj, I think. No vessel required a ligature.

The operation was done with the patient in the sitting posture, under the influence of chloroform; the head being supported by an assistant.

On account of the still rather feeble condition of the patient, it was deemed unsafe to make a profound anæsthetic impression upon him with chloroform; consequently the operation was frequently interrupted by the struggles of the patient, and delayed by the repeated administration of the anæsthetic. Notwithstanding the struggles of the patient, he had no recollection of the operation when it was concluded; and shock to the nervous system was effectually prevented.

On the sixth day after the operation the sutures were all removed, and the wound found healing by first intention, except half an inch beneath the external angle of the eye, where apposition had been disturbed by the dragging downwards of the cheek.

For two or three days, commencing about the second day after the operation, the patient had some fever, and complained of some pain in the head ; but made a rapid recovery, and about the ninth or tenth day was walking about the yard.

There was difficulty in deglutition after the operation, but much less than I anticipated. There was also great difficulty in articulation, but it gradually became better, and after the adjustment of a gold plate, by Dr. H. Lawrence, the boy articulated very well.

I was informed a few days since, by Judge Hill, that the boy's health was unexceptionable. So far there seems to be no disposition to a return of the disease.

As to the character of the tumor, I cannot pronounce confidently. The bones had been absorbed to a great extent, leaving little more than mere shells or scales. The tumor seemed partly granular, and partly fibrous, with an admixture of fat. In its removal, I cut, in the language of Mr. Liston, "beyond the disease."

The incision through the lip was done along the line bounding the sulcus, thinking that the deformity would be less. Whether it is or not, I am unable to say : but the *extent* of the cut was somewhat less than it would have been had it been carried beneath the column of the nose, thence through the sulcus.

For the purpose of stuffing the cheek after this operation, a thin gum-elastic bag, arranged so that it might be inflated after its introduction, would be an improvement on the present plan of stuffing with lint. In its flaccid state, it would be easy of introduction ; and, having discharged the air from it, it could be easily removed—while its texture would prevent it being caught on any salient point of bone. Besides these advantages, it would not absorb the secretions of the mouth and cut surface; and, consequently, would be more cleanly and comfortable.

In performing the dissection beneath the globe of the eye, I derived advantage from the use of an instrument, by which this organ was shielded from danger, and at the same time held up, so that I could see how and where to cut. The instrument was made by an ingenious blacksmith, after a model cut from pasteboard, and moulded, while wet, to fit the floor of the orbit. It is of silver; in shape much like a teaspoon, with its bowl truncated, and the handle curved in the shank, so that it stands at nearly a right angle with the bowl, having its extremity curved, so as to rest securely over the thumb. It is held between the thumb and first two fingers of the hand, while the remaining fingers rest firmly on the forehead of the patient; the assistant inserts the truncated bowl beneath the globe, as the tissues are divided by the knife.—*Am. Jour. Med. Sciences.*

